# The first prevalence and antifungal susceptibility profile of *Candida* infections in Palestine, 2022

**DOI:** 10.1186/s12879-024-10062-3

**Published:** 2024-10-11

**Authors:** Hanaa Baniodeh, Rasmi Abu-Helu, Mohand Abulihya, Mohammed Y. Awwad, Ayman Dawoud, Faiza Tebbji, Adnane Sellam

**Affiliations:** 1 Department of Medical Laboratory Sciences, Faculty of Health Professions, Al-Quds University, Jerusalem, Palestine; 2 Department of Pathology, Al Istishari Arab Hospital, Ramallah, Palestine; 3 Infection Control Office, Al Istishari Arab Hospital, Ramallah, Palestine; 4 Department of Pathology, An-Najah National University Hospital, Nablus, Palestine; 5 Montreal Heart Institute/Institut de Cardiologie de Montréal, Université de Montréal, Montréal, Québec Canada; 6 Department of Microbiology, Infectious Diseases and Immunology, Faculty of Medicine, Université de Montréal, Montréal, Québec Canada

**Keywords:** *Candida albicans*, Epidemiology, Antifungal susceptibility, Non-*albicans Candida* Spp

## Abstract

**Background:**

*Candida* spp. are the most common cause of opportunistic fungal infections and are associated with a high mortality rate worldwide. In Palestine, the prevalence of *Candida* spp. infections remains elusive.

**Methods:**

We performed our study at two hospitals in Palestine (Istishari Arab Hospital, and Najah National University Hospital). All patients diagnosed with candidiasis during the year 2022 have participated in the study. The prevalence of *Candida* spp., their distribution, and the activity of selected antifungals against *Candida* pathogens were assessed. In combination with phenotypic properties, *Candida* isolates were identified and tested for antifungal susceptibility using the colorimetric VITEK-2 Compact system.

**Results:**

Our results showed that the prevalence of *Candida* spp. among infected samples was 11.6%. A total of eleven different *Candida* spp. were identified. Among these isolates, *C. albicans* (46.54%) was the most frequent, followed by *C. glabrata* (16.14%), *C. tropicalis* (13.83%), *C. parapsilosis* (4.82%), *C. krusei* (3.56%), *C. dubliniensis* (2.09%), *C. ciferrii* (1.67%), *C. lusitaniae* (0.83%), *C. guilliermondii* (0.62%), *C. kefyer* (0.41%) and *C. spherica* (0.20%). Among *C. albicans*, all isolates were 100% susceptible to fluconazole and micafungin. The susceptibility rates to Amphotericin B and flucytosine were 95% and 99%, respectively. The susceptibility rates of non-*albicans Candida* spp. (NAC) to fluconazole, voriconazole, amphotericine B, caspofungin, flucytosine and micafungin were 70%, 99%, 97%, ,72%, 92% and 100%, respectively. The incidence of *Candida* infections was higher in the intensive care unit and surgery department as compared to other hospital departments.

**Conclusions:**

Four pathogens are responsible for the most invasive infections: *C. albicans*, *C. glabrata*, *C. tropicalis*, and *C. parapsilosis*. A notable characteristic of this study was the high frequency of NAC species which were often more resistant to antifungal agents. A quick and accurate system like Vitek 2 compact was suggested for the careful species identification of clinical isolates of *Candida*. We suggest that continued surveillance of species distribution and susceptibility to antifungals will enhance future burden estimates and assist in evaluating preventative measures’ effectiveness.

## Introduction

Over the past 30 years, there has been a significant increase in fungal infections in humans [1]. The members of the genus *Candida* are most frequently recovered from human fungal infection [2]. Currently, there are more than 100 heterogeneous species that belong to the *Candida* genus and only 17 different species are known to be aetiological agents of human and animal infections [2]. Species including *Candida albicans*,* C. glabrata*,* C. tropicalis*,* C. parapsilosis*, and *C. krusei*, are responsible for over 90% of all invasive candidiasis [3]. *C. albicans* is the primary cause of candidiasis and is responsible for about 80% of all *Candida* infections [3]. However, Non-*albicans Candida* (NAC) species such as *C. glabrata*,* C. tropicalis*, and *C. parapsilosis* are also frequent causes of candidemia and their frequency is increasing in many geographical areas [3]. As commensal organisms, *Candida* spp. predominantly colonize the gastrointestinal and urogenital tracts of healthy humans. However, many *Candida* species are important opportunistic pathogens especially in immunocompromised patients [4]. Fungal infection caused by *Candida* spp. can be either superficial, affecting the skin, or it may be deep, affecting the joints, hair, nails, and mucosal membranes, or even systemic, which affects major organs and can be life-threatening if not treated promptly [5]. Both *C. albicans* and *C. glabrata* are natural components of the vaginal fungal microbiota and are the main cause of vulvovaginal candidiasis (VVC) which affects 70–75% of childbearing women at least once, and 40–50% of them will experience recurrence [6].

The significantly increased emergence of *Candida* spp. as human pathogens can be attributed to enhanced identification techniques [7]. Regarding variations in *Candida* spp. susceptibilities to antifungal medications, identification of isolates to the species level is crucial for prompt and effective treatment [8, 9]. Antifungal treatment depends on the type of infection, anatomical site, and sensitivity profile of *Candida* species [10]. Therapeutic options are limited to treatment with mainly five antifungal classes, namely polyenes, azoles, fluoropyrimidine, echinocandins, and allylamines [11].

As species and antifungal susceptibility profiles of *Candida* differ among geographic areas, it is thus of high importance to assess the prevalence of *Candida* spp. The aim of the current study is to gain a better understanding of contemporary trends in *Candida* spp. distribution and resistance patterns, which would guide diagnosis and prompt therapy. To our knowledge, this is the first comprehensive investigation of the prevalence of *Candida spp.* and their antifungal susceptibility in Palestine. While former studies focused on specific *Candida* infections (e.g. VVC [12–14], cutaneous infections [13]) or hospital ward (e.g. oncology [15]), our work examined *Candida spp.* epidemiology in different care facilities in two large hospitals (Istishari Arab Hospital and Najah National University Hospital) during one year.

## Methods

### Data and sample collection

Our study examined the prevalence of *Candida* species among patients admitted to two hospitals in Palestine: Istishari Arab Hospital and Najah National University Hospital. The two Hospitals receive patients from all parts of the West Bank and Gaza Strip. Participants in this study were patients admitted to the two hospitals in 2022 for various reasons. The study focused on patients who had samples tested in the microbiology laboratory and received a positive culture result. The clinical samples included urine, blood, vaginal swabs (both high and low), tip culture, respiratory tract samples (sputum, bronchial wash, bronchial aspiration lavage, and throat), fluid samples (synovial, pleural, peritoneal, CSF, drain, and semen), and other swab cultures (wound, abscess, pus, eye, ear, and breast).

### Identification of *Candida* species

In this study, the identification of *Candida* spp. was carried out by the two hospitals through the implementation of the following steps: First of all, clinical samples were cultured on Blood and MacConkey agar (HiMedia, India) and incubated at 37 °C for 48 h. The process of identification started with traditional techniques, including gram staining (Química Clínica Aplicada, Spain), and assessing the morphological characteristics of colonies. The HiCrome *Candida* Differential Agar Base (HiMedia, India) was used to quickly isolate and identify *Candida* species including *C. albicans*,* C. krusei*,* C. tropicalis*,* and C. glabrata*.

The VITEK2 Compact system was used to precisely detect *Candida* species. To begin the process, an inoculum from a pure culture was taken. Homogeneous suspension of yeast cells with a density equivalent to a McFarland No. 1.80 to 2.20, was analyzed using a calibrated VITEK^®^ 2 DensiCHEK™ Plus. The VITEK2 YST ID card (bioMérieux, France) was used to identify *Candida* species.

### Antifungal susceptibility assays

Antifungal susceptibility tests were done at the two hospitals using the Vitek 2 system (AST-YS08 card; bioMérieux, France). The test was performed according to the clinical and Laboratory Standards Institute (CLSI) breakpoints (M27M44S) and included fluconazole, voriconazole, micafungin, caspofungin, amphotericin B, and flucytosine.

### Ethics

This study was conducted in the Department of Microbiology at Istishari Arab Hospital and Najah National University Hospital. The study underwent approval by the Research Ethical Committee from Al-Quds University to ensure its conformity with ethical standards. The hospitals involved in the study were informed about the study’s goals and objectives and were required to fill out a consent form before participating.

## Results

### Patient characteristics and prevalence of *Candida* infections

**Fig. 1:**
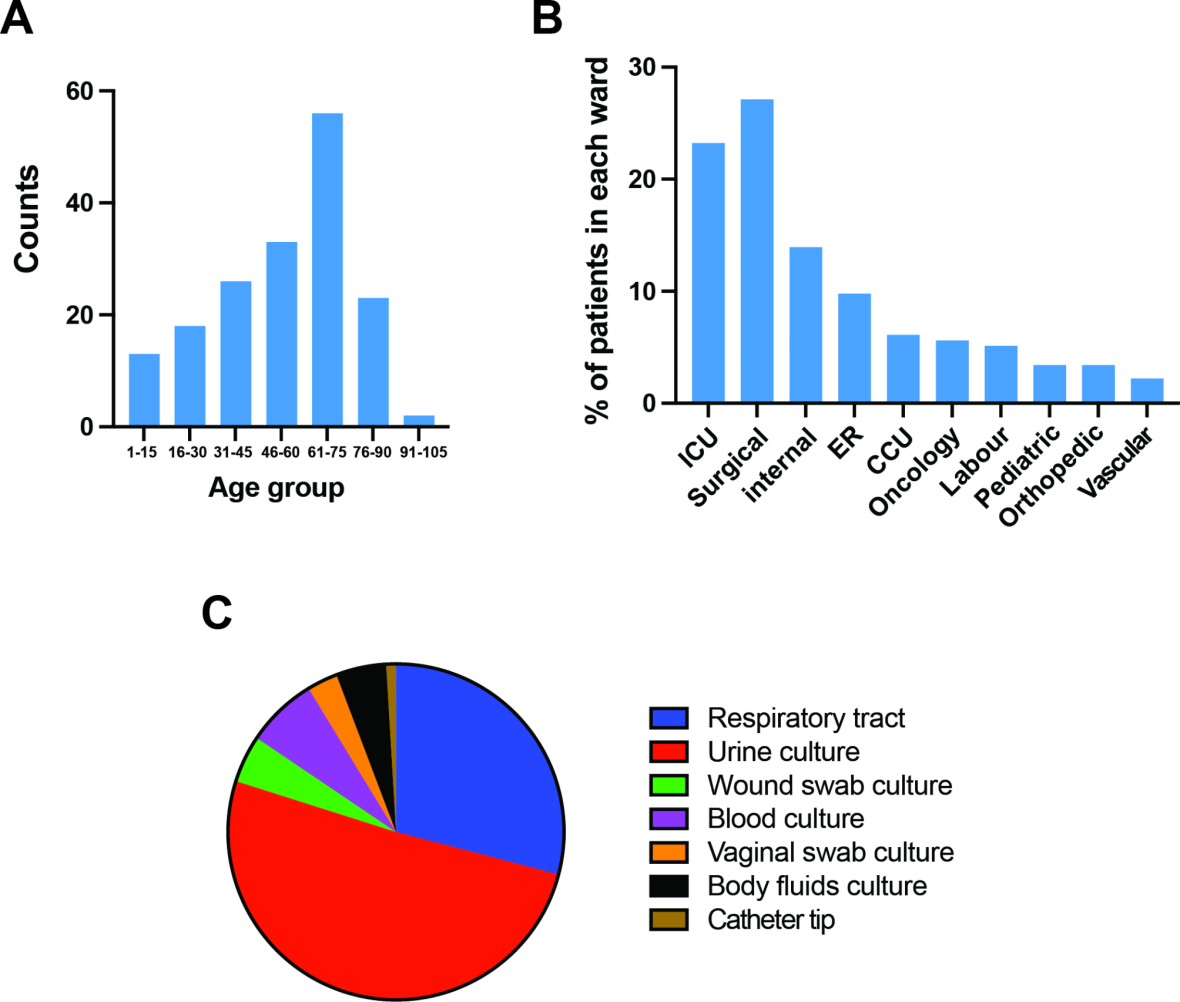
Age and ward distribution of patients. (**A**) Age distribution of patients enrolled in this study. (**B**) Distribution of patients in different hospital wards. Values are the percentage of patients per hospital departments. ICU: Intensive Care Unit; ER: Emergency Room; CCU: Critical Care Unit. (**C**) Prevalence of *Candida* infections in samples taken from IAH and NNUH. Data are represented as the percentage of *Candida* spp. infection for each sampling site relative to the total *Candida* infection (*n* = 746)

A total of 410 patients (204 females and 206 males) diagnosed with sporadic or reccurent Candidiasis were enrolled from the Istishari Arab (IAH) and Najah National University (NNUH) hospitals, representing 12.3% (746 *Candida* infections /6059 total infection cases) of the overall patients recorded with infections. The age of patients with *Candida* infections ranged from 1 to 91 years with an average of 53.20 years (female: 50.72 and male: 56.16). The largest group of patients fell within the age bracket of 61 to 75 years old (Fig. 1A; Table 1).

**Table 1:** Age, sex and hospital ward distribution of patients at IAH and NNUH hospitals

		number	%
Sex			
	Female	204	49.8
	Male	206	50.2
Hospital ward			
	ICU	95	23.2
	Surgical	82	20.1
	Internal	57	14
	ER	40	9.8
	CCU	25	6.1
	Oncology	23	5.6
	Labour	21	5.2
	Pediatric	14	3.4
	Orthopedic	14	3.4
	Vascular	9	2.2
Age			
	1–15	13	7.6
	16–30	18	10.5
	31–45	26	15.2
	46–60	33	19.3
	61–75	56	32.8
	76–90	23	13.4
	91+	2	1.2

Occurrence of *Candida* infections among patients differed across the hospital wards. The surgical department has the highest percentage of patients diagnosed with candidiasis, accounting for 27.1% of the total cases. Following closely is the intensive care unit (ICU) department, with 23.2% of patients diagnosed with *Candida* infections. The internal department accounted for 13.9% of cases, while the ER had 9.8% of patients with positive *Candida* cultures. The critical care unit (CCU) had 6.1%, oncology accounted for 5.6%, labour had 5.1%, and orthopedic had 3.4% of cases. The pediatric department also had 3.4% of cases, while the vascular department had the lowest percentage at 2.2% (Fig. 1B; Table 1).

Many patients enrolled in this study experienced different episodes of *Candida* infections. Of the 410 patients, 135 (32%) had recurrent *Candida* infections, occurring once or multiple times. This can be attributed to some patients being infected with either the same or different *Candida* spp. isolate. Overall, a total of 749 cases of *Candida* infections were recorded out of the entire 6059 infections in the two hospitals which reflect a high prevalence rate (123 per 1000 infections). This suggests that *Candida* infections remain a significant concern in these healthcare settings and highlights the need for continued monitoring and prevention efforts. We also found that urinary tract samples had the highest *Candida* infection rate at 50.8%, followed by respiratory samples at 29.08% (Fig. 1C; Table 1).

### Prevalence of *Candida* species

**Fig. 2:**
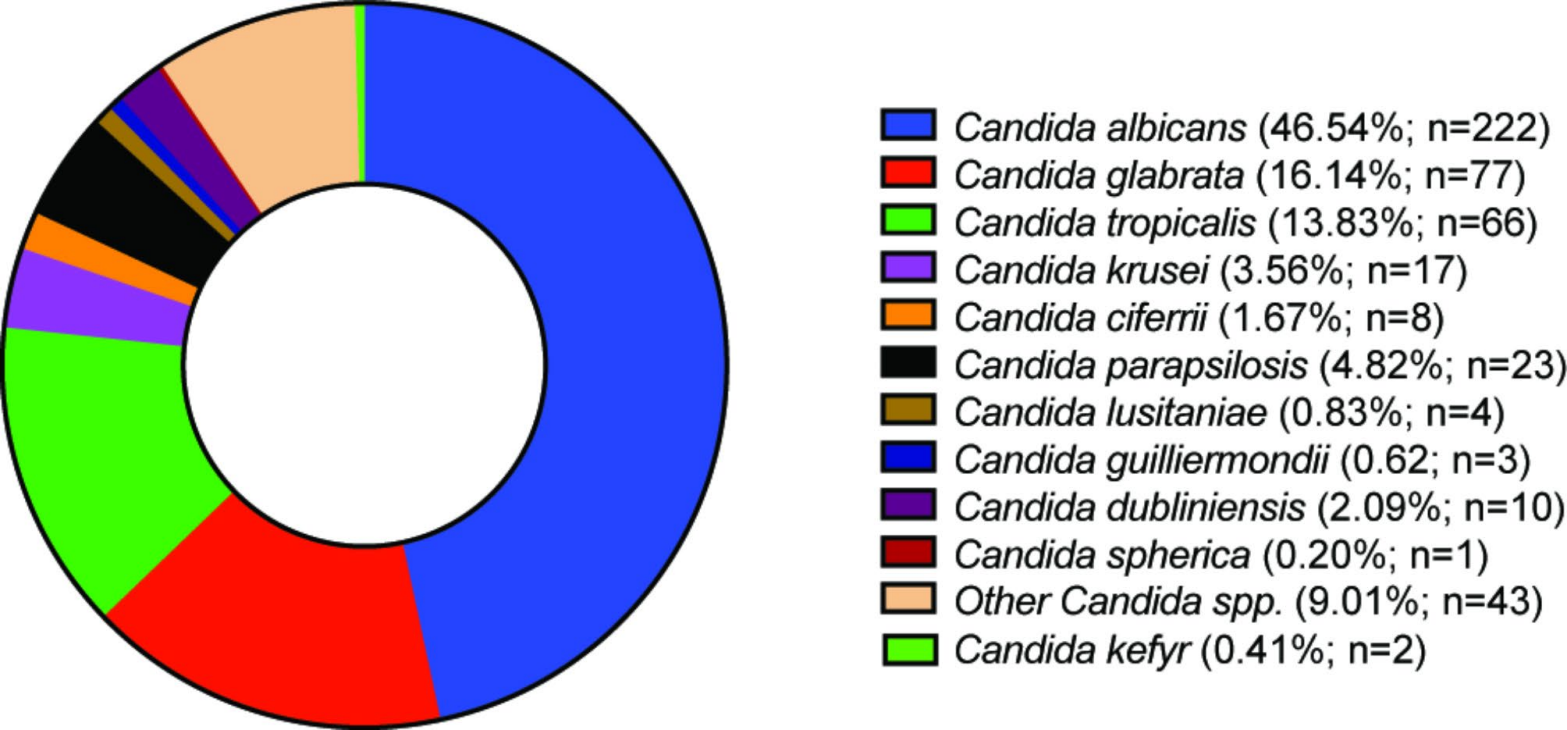
Distribution of ***C. albicans*** and non-*albicans Candida* species in samples collected from the IAH and NNUH. A total of 476 *Candida* spp. were identified by chromogenic *Candida* agar and Vitek2 YST identification card

We analyzed the 749 cases of *Candida* infections from the 410 enrolled patients for species prevalence. Eleven distinct *Candida* spp. were identified among these isolates. *C. albicans* (46.54%) was the most frequent, followed by *C. glabrata* (16.14%), *C. tropicalis* (13.83%), *C. parapsilosis* (4.82%), *C. krusei* (3.56%), *C. dubliniensis* (2.09%), *C. ciferrii* (1.670%), *C. lusitaniae* (0.83%), *C. guilliermondii* (0.62%), *C. kefyer* (0.41%) and *C. spherica* (0.2%) (Fig. 2).

### Antifungal susceptibility patterns of *Candida* species

**Fig. 3:**
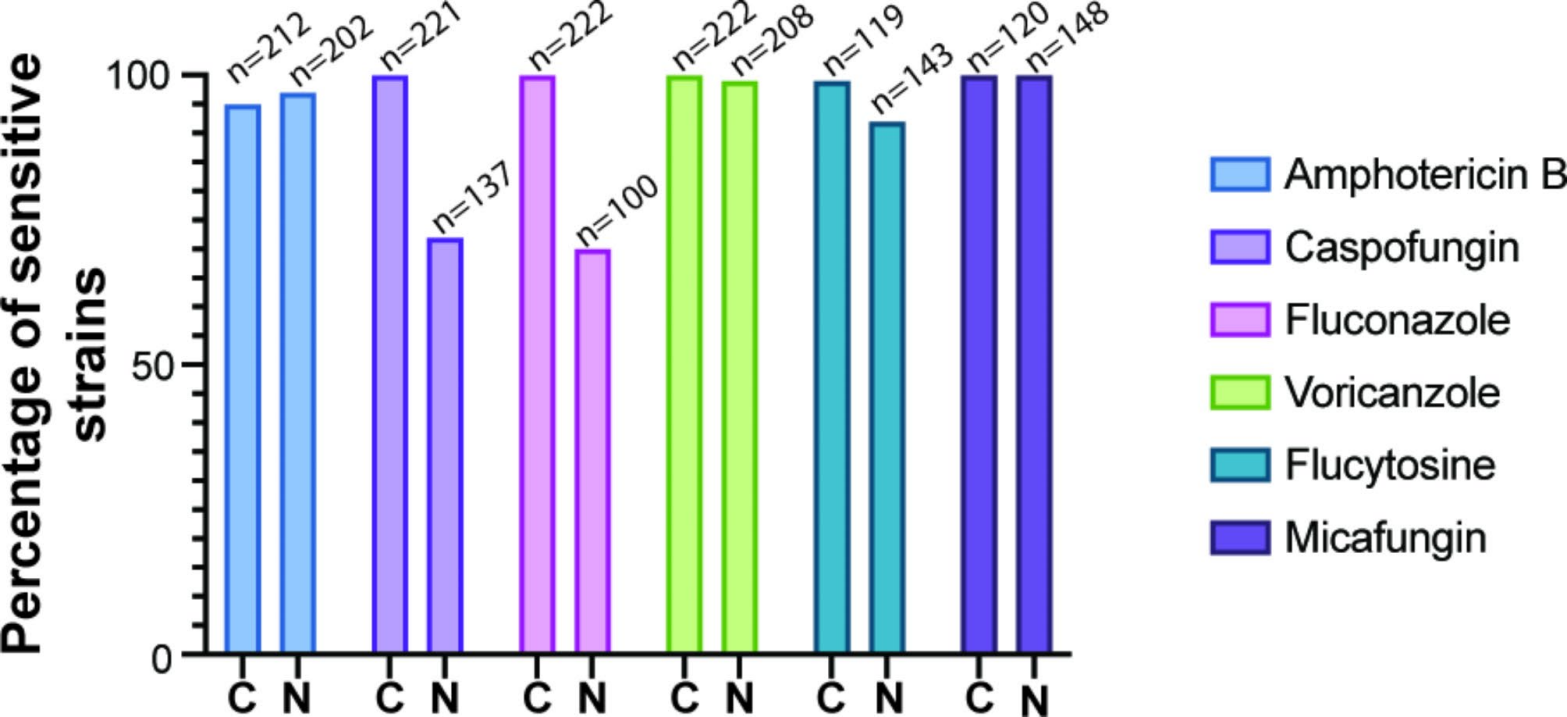
Sensitivity rate of *C. albicans* and non-*albicans Candida* isolates in samples collected from the IAH and NNUH. The susceptibility of 432 isolates of *C. albicans* (C) and non-*albicans Candida* (N) to standard antifungal agents (amphotericin B, fluconazole, voriconazole, flucytosine, micafungin and caspofungin) were assessed

The susceptibility of 432 isolates of *Candida* spp. to antifungal agents, including amphotericin B, fluconazole, voriconazole, flucytosine, micafungin, and caspofungin, was determined and data are summarized in Table 2. The majority of the tested *Candida* spp. isolates were sensitive to amphotericin B, flucytosine, and micafungin. *C. albicans* isolates were highly sensitive to all antifungal classes with a susceptibility rate of over 95%. Out of the 30 *C. glabrata* isolates, only nine were sensitive to fluconazole. Regarding caspofungin sensitivity, 22% of *C. glabrata* isolates were resistant, 40% were sensitive, and 37% were intermediate. With the exception of one *C. tropicalis* isolate that was resistant to flucytosine, all others were found to be sensitive to all antifungal agents. All *C. krusei* isolates were resistant to fluconazole and flucytosine, with only 16% showing sensitivity to caspofungin. Additionally, the four tested isolates of *C. lusitaniae* were resistant to amphotericin B. Overall, we found that NAC species exhibited elevated resistance levels to caspofungin and fluconazole as compared to *C. albicans* (Fig. 3).

**Table 2:** Antifungal susceptibility pattern of *Candida* species isolated at IAH and NNUH hospitals. Of note, for some antifungals, only a fraction of *Candida* spp. isolates was tested. R: resistant; S: sensitive; I: intermediate

Antifungal	Amphotericin B			Caspofunagin			Fluconazole			Voricanzole			Flucytosine			Micafunagin		
	R	S	I	R	S	I	R	S	I	R	S	I	R	S	I	R	S	I
*C. albicans*	8 (3.6%)	212	2	1 (0.5%)	221	0	0	222	0	0	222	0	0	119	1	0	120	0
*C. glabrata*	0	76	0	17 (22%)	31	29	21 (70%)	9	0	2 (2.6%)	74	0	0	66	0	0	65	0
*C. tropicalis*	0	67	0	0	67	0	0	67	0	0	67	0	1 (2%)	50	0	0	51	0
*C. krusei*	0	16	2	1 (5.5%)	3	14	10 (100%)	0	0	0	18	0	11 (100%)	0	0	0	11	0
*C. ciferrii*	1 (12.5%)	6	1	4 (50%)	4	0	8 (100%)	0	0	0	8	0	0	1	0	0	1	0
*C. parapsilosis*	0	23	0	0	23	0	7 (30%)	16	0	0	23	0	0	18	0	0	18	0
*C. lusitaniae*	4 (100%)	0	0	0	4	0	0	4	0	0	4	0	0	1	0	0	1	0
*C. guilliermondii*	0	3	0	0	4	0	1 (33.3%)	2	0	0	3	0	0	1	0	0	1	0
*C. dubliniensis*	0	9	0	0	1	0	0	2	0	0	9	0	1 (14%)	6	0	0	0	0
*C. kefyr*	0	2	0	0	0	0	0	0	0	0	2	0	0	0	0	0	0	0
Total	**13** (3%)	**414**	**5**	**23** (5.4%)	**358**	**43**	**47** (12.7%)	**322**	**0**	**2** (0.5%)	**430**	**0**	**13** (4.7%)	**262**	**1**	**0** (0%)	**268**	**0**

## Discussion

In this study, and for the first time in Palestine, we assessed the prevalence of *Candida* infections in two large hospitals for one year (2022). Our goal was to gain a better understanding of contemporary trends in *Candida* spp. distribution and resistance patterns, which could inform treatment decision-making. Due to the significant geographical variance in these factors, our analysis aimed to provide valuable insights for healthcare professionals.

According to this study, there are significant variations in the rates of *Candida* infection among different hospital departments. The highest percentage of patients diagnosed with Candidiasis was found in the surgical department, followed closely by ICU which is similar to what was observed in many countries in Europe where *Candida* bloodstream infections were commonly associated with the same hospital wards [16]. These findings underscore the need for hospitals to implement proper monitoring and prevention measures to reduce the incidence of Candidiasis infections, especially in departments like ICU and surgery. To accomplish this, hospitals should establish regular monitoring programs to identify and address potential sources of infection. They should also educate both patients and staff on the importance of maintaining proper hygiene and infection control practices to prevent the spread of *Candida* infections. We found that respiratory tract samples demonstrated the second highest rate of *Candida* infection at 29.08%, behind urine samples (50.08% infection rate). *Candida* spp. such as *C. albicans* are natural colonizers of the oral cavity, gastrointestinal mucosa, and respiratory tract. Thus, a fraction of the identified *Candida* isolates in respiratory tract could originate from sample contamination by commensal *Candida* yeasts. Since there is no method that can discriminate between commensal and infectious lifestyle of *Candida* spp., the high rate of occurrence of these yeasts in respiratory tract samples should be interpreted with high caution [17].

The findings of the study revealed that among various species of *Candida*, *C. albicans* was the most frequently observed, accounting for almost half of the distribution (46.54%). The relative proportion of *C. albicans* observed is similar to what was reported in many counties all around the world [18–24] (Table 3). However, a considerable proportion of NAC species were also identified. Among them, *C. glabrata* was the predominant species with a prevalence rate of 16.14%, followed by *C. tropicalis* (13.83%) and *C. parapsilosis* (4.82%). Overall, our study underscored a conserved epidemiological trend with *C. albicans* being the dominant species followed by *C. glabrata* and *C. tropicalis* as the most frequent *Candida* spp. among NAC.

**Table 3:** Candida spp. distribution in several studies

Reference	C. albicans	C. glabrata	C. tropicalis	C. parapsilosis
**This study**	46.54%	16.14%	13.83%	4.82%
Kajihara et al. [25]	43.6%	19.5%	6.7%	18.8%
Badiee et al. [26]	47.7%	20.9%	3.8%	10%
Hu et al. [27]	75.3%	2.02%	6.06%	-
Jayant et al. [28]	34.07%	16.29%	27.4%	5.92%
Bilal et al. [18]	49.36%	11.37%	21.89%	13.92%
Pfaller et al. [19]	45.3–57.4%	15.9–19.6%	8.3–10.7%	12.3–17.6%
Isreal et al. [29]	39.4%	18.8%	18%	14.4%
Remington et al. [20]	48%	32%	4%	5.2%
Ricotta et al. [21]	48%	24%	6%	7%
Khan et al. [30]	37.22%	10.2%	14.5%	34.66%

*C. albicans* is thought to be intrinsically susceptible to all antifungal drugs, and resistance can develop solely through acquisition [31]. Our research findings support this postulate as the majority of *C. albicans* isolates were highly responsive to all tested antifungals. This aligns with previous studies on the subject and reinforces the understanding that *C. albicans* is not inherently resistant to antifungal drugs.

As the occurrence of *Candida* infection rises, there is a noticeable shift towards a larger percentage of NAC. These species such as *C. krusei* are often naturally resistant to fluconazole [32]. Our findings indicate that all *C. krusei* strains are resistant to fluconazole. Unlike other opportunistic *Candida* spp., *C. lusitaniae* has been observed to develop resistance towards the commonly used antifungal agent, amphotericin B [33]. Our research findings indicate that all the tested isolates were resistant to amphotericin B. Such observations highlight the need for alternative treatment options. It is believed that certain types of *C. glabrata* are naturally resistant to fluconazole and itraconazole [7]. Our research findings confirmed that *C. glabrata* has the lowest susceptibility rate of 30% to fluconazole. However, we observed a high level of susceptibility to amphotericin B and voriconazole at 100% and 97%, respectively, which is consistent with existing literature [7].

According to this study, *C. parapsilosis* exhibits a low susceptibility rate to fluconazole at 69.5%, which is consistent with the findings of a similar study conducted in Jerusalem, which recorded a rate of 62.2% [29]. It is worth noting that this species is increasingly becoming prevalent [19], and it can be transmitted through hand carriage in healthcare settings, thus increasing the likelihood of catheter-related Candidemia [34]. This emphasizes the need for proper hygiene and infection control in healthcare facilities to avoid the spread of this potentially dangerous infection. The findings in Table 2 indicate that NAC spp. exhibited significantly greater resistance to all antifungal agents when compared to *C. albicans*. This observation is consistent with different former studies listed in Table 4. Consequently, treating NAC spp. may prove to be more challenging and require additional research to elucidate the underlying causes of their heterogeneous resistance pattern.

**Table 4:** Antifungal susceptibility rate for *C. albicans* and NAC in several studies

Reference	Setting	Candida spp.	FLU^*^	AmB^*^	VOR^*^	5-FC^*^
This Study	All clinical samples	*C. albicans*	100%	95%	100%	99%
		NAC	70%	97%	99%	92%
Černáková et al. [35]	Oral candidiasis	*C. albicans*	92%	100%	100%	42%
		NAC	28%	100%	71%	57%
Kajihara et al. [25]	Candiduria	*C. albicans*	98.9%	98.5%	99%	-
		NAC	88.3%	95.2%	90.9%	-
Toner et al. [36]	Candiduria	*C. albicans*	93%	100%	98%	94%
		NAC	65%	100%	75%	92%
Isreal et al. [29]	Candidemia	*C. albicans*	96.7%	100%	97.8%	-
		NAC	53%	98.1%	55.3%	-

(*FLU: Fluconazole; AmB: Amphotericin B; VOR: Voriconazole; 5-FC: flucytosine)

## Conclusion

Through our analysis, we found that *C. albicans*,* C. glabrata*,* and C. tropicalis* were the most common types of *Candida*, representing roughly 90% of all the cultured *Candida*. *C. albicans* is a major cause of Candidiasis, and antifungal resistance remains low. In line with other global studies, the prevalence of non-*albicans* candidiasis was higher. We found a high rate of fluconazole non-susceptibility among NAC. Our study also reported a high rate of caspofungin resistance among *C. glabrata*, which is a matter of concern. It is crucial to continually monitor the epidemiology and antifungal resistance of *Candida* isolates in various geographic regions and large urban areas to track clonal patterns with concordant resistance mechanisms. This will enable a better selection of empiric antifungal therapy and ultimately contribute to better patient outcomes.

### Study limitation

Our study has some limitations as it was conducted retrospectively. Hence, we could not identify any specific risk factors for a higher prevalence of *Candida* infection. We faced the challenge of not having clinical data correlating with microbiology results, as the samples may have been taken from symptomatic or asymptomatic patients. Furthermore, our research was conducted in only two hospitals in Palestine; therefore, the results may not be applicable to other district general hospitals.

## Data Availability

All data related to this study are presented in the text and tables of the manuscript.

## Abbreviations

NAC: Non-albicans Candida
ICU: Intensive Care Unit
ER: Emergency Room
CCU: Critical Care Unit. IAH: Istishari Arab hospital
NNUH: Najah National University hospital
